# Flexibility and Bed Margins of the Community of Madrid’s Hospitals during the First Wave of the SARS-CoV-2 Pandemic

**DOI:** 10.3390/ijerph18073510

**Published:** 2021-03-28

**Authors:** Eugenio F. Sánchez-Úbeda, Pedro Sánchez-Martín, Macarena Torrego-Ellacuría, Ángel Del Rey-Mejías, Manuel F. Morales-Contreras, José-Luis Puerta

**Affiliations:** 1 Institute for Research in Technology (IIT), ICAI School of Engineering, Comillas Pontifical University, 28015 Madrid, Spain; psanchez@comillas.edu (P.S.-M.); mfcontreras@icade.comillas.edu (M.F.M.-C.); 2 Unidad de Innovación, Hospital Clínico San Carlos, IdISSC, 28040 Madrid, Spain; macarena.torrego@salud.madrid.org (M.T.-E.); alreymejias@gmail.com (Á.D.R.-M.); 3 Departamento de Psicobiología y Metodología en Ciencias del Comportamiento, Facultad de Psicología, Universidad Complutense, 28223 Madrid, Spain; 4 Faculty of Business Management and Economics, ICADE, Comillas Pontifical University, 28015 Madrid, Spain; 5 Consejería de Sanidad y Dirección General de Estadística, Comunidad de Madrid, 28013 Madrid, Spain; puerta.lopez-cozar@salud.madrid.org

**Keywords:** hospital bed management, flexibility, bed margin, intensive care, non-intensive care, coronavirus, COVID-19

## Abstract

*Background:* The COVID-19 pandemic has had global effects; cases have been counted in the tens of millions, and there have been over two million deaths throughout the world. Health systems have been stressed in trying to provide a response to the increasing demand for hospital beds during the different waves. This paper analyzes the dynamic response of the hospitals of the Community of Madrid (CoM) during the first wave of the severe acute respiratory syndrome coronavirus 2 (SARS-CoV-2) pandemic in the period between 18 March and 31 May 2020. The aim was to model the response of the CoM’s health system in terms of the number of available beds. *Methods:* A research design based on a case study of the CoM was developed. To model this response, we use two concepts: “bed margin” (available beds minus occupied beds, expressed as a percentage) and “flexibility” (which describes the ability to adapt to the growing demand for beds). The Linear Hinges Model allowed a robust estimation of the key performance indicators for capturing the flexibility of the available beds in hospitals. Three new flexibility indicators were defined: the Average Ramp Rate Until the Peak (ARRUP), the Ramp Duration Until the Peak (RDUP), and the Ramp Growth Until the Peak (RGUP). *Results:* The public and private hospitals of the CoM were able to increase the number of available beds from 18,692 on 18 March 2020 to 23,623 on 2 April 2020. At the peak of the wave, the number of available beds increased by 160 in 48 h, with an occupancy of 90.3%. Within that fifteen-day period, the number of COVID-19 inpatients increased by 200% in non-intensive care unit (non-ICU) wards and by 155% in intensive care unit (ICU) wards. The estimated ARRUP for non-ICU beds in the CoM hospital network during the first pandemic wave was 305.56 beds/day, the RDUP was 15 days, and the RGUP was 4598 beds. For the ICU beds, the ARRUP was 36.73 beds/day, the RDUP was 20 days, and the RGUP was 735 beds. This paper includes a further analysis of the response estimated for each hospital. *Conclusions:* This research provides insights not only for academia, but also for hospital management and practitioners. The results show that not all of the hospitals dealt with the sudden increase in bed demand in the same way, nor did they provide the same flexibility in order to increase their bed capabilities. The bed margin and the proposed indicators of flexibility summarize the dynamic response and can be included as part of a hospital’s management dashboard for monitoring its behavior during pandemic waves or other health crises as a complement to other, more steady-state indicators.

## 1. Introduction

When severe acute respiratory syndrome coronavirus 2 (SARS-CoV-2) was first discovered in Wuhan (China) in December 2019, even the most qualified experts did not anticipate that it would rapidly spread to create the worst global public health crisis since the 1918 flu pandemic [1]. The total number of COVID-19 cases in the world is counted in the tens of millions, with deaths in over two million cases. It is well known that emerging viral pandemics “can place extraordinary and sustained demands on public health and healthcare systems and on providers of essential community services” [2]. A crisis such as the current COVID-19 pandemic is a dramatic event, which takes place in a difficult environment with enormous emotional tension, coupled with a serious disproportion between needs and available resources. In these circumstances, managing healthcare services is a constant and changing challenge [3]. Therefore, flexibility in the usage of hospital beds is a crucial element for efficiently organizing critical capacity [4]. In Spain, as in many other countries, in the spring of 2020, COVID-19 produced such a significant number of seriously ill patients that the healthcare system almost collapsed. In some of its Autonomous Communities (ACs), particularly in the Autonomous Community of Madrid (CoM), where the capital of Spain (Madrid city) is located, the situation was especially pronounced.

Thus, on 31 March, at least 450,611 cases had been reported in Europe. The CoM registered a cumulative incidence in the previous 14 days of 363.22 and Spain registered 192.3, followed by Italy (122.2), Belgium (103), France (56.6), and the UK (40). The accumulated cases in the CoM were 29,840, 29% those of all of Spain (102.136). The highest fatality rate in Europe was in Italy (11.7), followed by Spain with 8.9, while in the CoM, it reached 13 (46% higher). In the CoM, the deaths per 100,000 inhabitants amounted to 57.7, tripling that of Spain (17.3), which was followed by Belgium (14.8), Italy (13.4), France (6.24), and the UK (5.6). The accumulated deaths in the CoM (3865) represented 42.7% of those registered in Spain (9053) [5,6]. If we look at the situation of beds in the CoM’s hospitals to describe their evolution, which is the main objective of this paper, it can be seen that the number of patients hospitalized just for COVID-19 in the intensive care units (ICUs) was 1514, and 13,713 patients were in beds other than intensive care (non-ICU). However, the situation can be described even better by comparing the figures of the CoM beds with those of Spain: 5872 (ICU) and 45,546 (non-ICU) [5]; Italy: 4023 (ICU) and 32,215 (non-ICU); France: 5496 (ICU) and 22,672 (non-ICU); and Belgium: 1088 (ICU) and 4989 (non-ICU) [7].

Due to the need to hospitalize a huge number of COVID-19 patients, which increased relentlessly with different degrees of severity during this first wave, the CoM’s hospitals rapidly increased their capacity to levels never seen before. Not only the number of hospital beds, but also the intensive support and invasive ventilation were of concern, as COVID-19 is a disease with a potentially fatal evolution. These circumstances meant that the number of available beds had to be increased from 18,692 (18 March 2020) to 23,623 (2 April 2020) in just 15 days, i.e., an increment of 26.4%. Although the first admission for COVID-19 in a CoM hospital occurred on 25 February 2020, in this paper, we analyze the data for the period between 18 March 2020 and 31 May 2020. The first date corresponds to the first day on which we had the complete data of the hospitals that are analyzed in this article.

The COVID-19 pandemic, as mentioned above, forced the CoM’s hospitals to increase the number of available beds. Given the shortage of papers in the world on the capacities of hospitals to increase their numbers of beds with the speed required by a pandemic wave, the aim of this article is to set dynamic performance indicators in order to model the dynamic response in terms of the number of available beds instead of using more stationary approaches. For this purpose, not only are specific visualization graphs used to show the daily evolution of the variables under consideration, such as the bed margin of the CoM’s hospitals, but a new set of indexes for quantifying their flexibility and a robust estimation method are proposed. Thus, the proposed approach to analyzing and quantifying the dynamics related to flexibility could be used for further analysis and as a reference for decision making not only at the hospital level, but also from a centralized or global perspective.

The remainder of this paper is structured as follows. Section 2 describes the materials and methods, and it is composed of three parts: (i) the healthcare system of the hospital network in the CoM; (ii) the data and the variables used in our study; and (iii) the methodology, where the proposed concepts of the bed margin and flexibility are defined. The results are presented in Section 3, first as an aggregate view of the healthcare system, and second as disaggregated view by hospital. Finally, Section 4 contains a discussion of the results, and conclusions and directions for future work are provided in Section 5.

## 2. Materials and Methods

### 2.1. The Hospital Network in the CoM

The Spanish National Health System (*Sistema Nacional de Salud*, SNS) is organized at two levels: national and regional. Its structure is a mirror of the administrative division of the country, i.e., the healthcare competences are transferred to the 17 ACs. The main actors in the ACs are the Departments of Health (*Consejerías de Sanidad*, CSs), which fulfill the role of a health authority, that is, healthcare delivery, regulation, planning, budgeting, and third-party payment. Above the CSs is the Spanish Ministry of Health, whose fundamental role, according to the law, is the coordination of the 17 CSs (through a collegiate body) and the publication of standards or laws that are mandatory throughout the SNS. In practice, CSs act with enormous autonomy, and there is little tradition of coordination among them [8].

The CoM’s public hospital network is made up of 34 hospitals, which are classified according to the degree of complexity of the medical procedures they offer (e.g., transplants, heart surgery, neurosurgery, radiotherapy, special treatments, etc.) into levels of high, medium, and low complexity, but only 28 of them were considered [9]. The other six are not acute-care general hospitals; therefore, they are of little interest for this study. However, we included a field hospital, Hospital IFEMA (HIFEMA), which began operating on 22 March 2020 and closed on 1 May 2020. In the CoM, although citizens can choose their public hospital and their primary care center, they are assigned a referral hospital depending on the geographic area in which they live. Furthermore, the referral hospitals of the primary care centers are the same as those of the patients under their care. Consequently, when citizens have to be hospitalized, they go to their referral hospital because of its proximity and, as in many cases, because they have been treated there on previous occasions. This explains why, if the geographical area that depends on a hospital has a high incidence of cases that require hospitalization, the hospital in question has a high occupancy.

In the period analyzed in this study, 33 private hospitals in the CoM also admitted COVID-19 patients, although for the purposes of this analysis, all of them were considered as a single hospital (HPRIVATE). This was due to the difference in the number of beds with respect to public hospitals (on 18 March 2020: 5885 vs. 12,807). This was also due to the fact that many of them were monographic hospitals and, above all, because hospital occupancy data were received aggregated as a group during most of the study period. Table 1 shows the main characteristics of the hospitals considered within this study. Note that the numbers of non-ICU and ICU beds were included as a mere reference of the sizes of the hospitals.

The Community of Madrid (CoM) is located in the center of Spain, with 6.7 million inhabitants. The population density varies within the community itself, with the vast majority of the population concentrated in the capital, Madrid, and its metropolitan area, making it one of the most densely populated regions in Europe. The population of Madrid, according to the latest 2019 census [10], is approximately 3.3 million. These inhabitants are concentrated in an area of 600 square kilometers. Thus, the population density of the capital city of Spain is 5500 persons per square kilometer. However, the appearance of cases does not only depend on population density, but also on other factors, e.g., mobility and social interaction.

**Figure 1:**
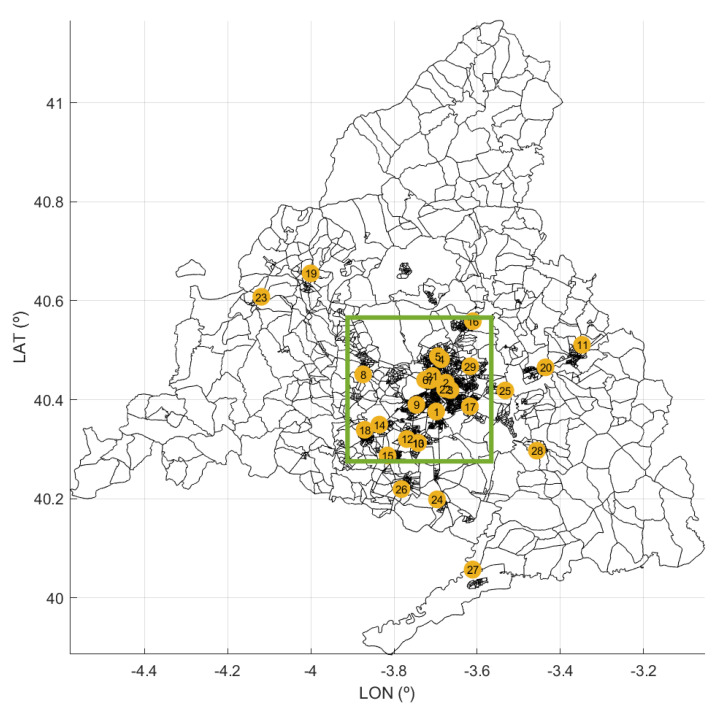
Map of the CoM with the locations of its public hospitals. The yellow tags correspond with the IDs in Table 1. Black patches represent regions with similar numbers of inhabitants.

**Figure 2:**
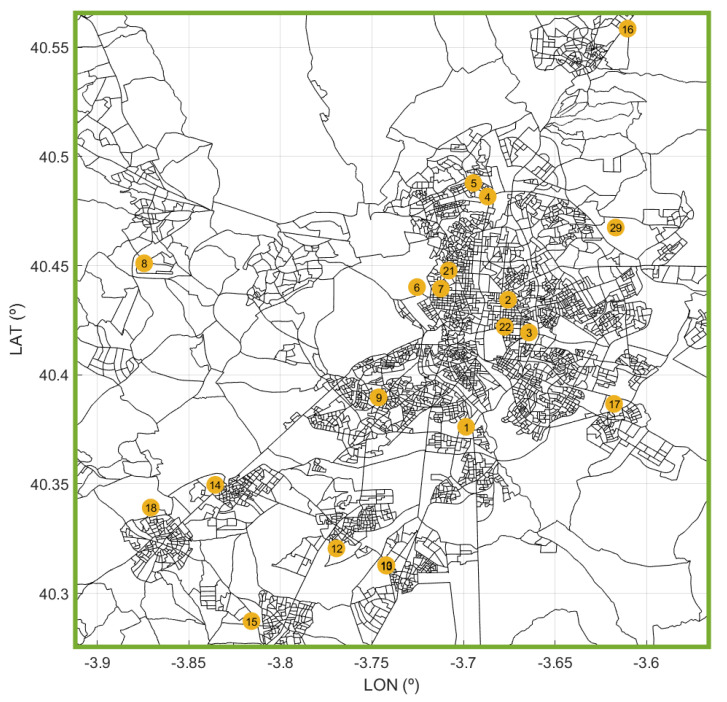
Zoom of Figure 1 with a detail of the metropolitan area of Madrid (green square in Figure 1).

The metropolitan region of Madrid follows a radio-centric spatial structure, which is similar to the organization that can be found in cities such as London or Paris. There is a central core form around which different areas have been developed, forming belts. Figure 1 shows the locations of the public hospitals of the CoM. Note that most of the hospitals are in the metropolitan area of the city (Figure 2). The hospital occupancy demand was heterogeneous in the CoM because the cumulative incidence of COVID-19 varied in the different geographical areas. The areas with the highest incidence in March and April were located in the periphery of the capital—the lower and upper quadrants of the area shown in Figure 2, specifically in the regions where hospitals 17, 10, 12, and 16 are located.

### 2.2. Data Description

It should be clarified that, during the pandemic, hospital beds were divided into two large groups (non-ICU beds and ICU beds); therefore, the collected data were ranked according to this classification. The beds assigned to each group were further subdivided into available beds (beds that had all the equipment and personnel necessary for their function), occupied beds (beds that were occupied by inpatients), and unoccupied beds (i.e., available beds that were not occupied). We also distinguished between the beds that were occupied by COVID-19 patients or non-COVID-19 patients. In pandemic waves, which pushed the CoM’s hospitals beyond their functional limits, it was necessary to organize the beds in a different way in order to control the situation as well as possible. Indeed, this not only happened in the CoM, but also in other countries. So, for example, the National Health Service of England wisely stated that the “hospital capacity has had to be organized in new ways as a result of the pandemic to treat COVID and non-COVID patients separately and safely... As a result caution should be exercised in comparing overall occupancy rates between this year and previous years” [11]. Bearing this advice in mind, this paper analyzes the evolution of beds in the CoM’s hospitals according to the structure and limits of the information received by the COVID-19 Control Center (CCC).

The data used in this study were supplied daily by each hospital, starting on 18 March 2020 and finishing on 31 May 2020. The original dataset consisted of 34 public and 33 private hospitals, although for the purposes of this study, we selected those described in Table 1. Notice that, although hospitals admitted COVID-19 patients prior to 18 March 2020, due to the lack of reliable data from hospitals during the beginning of the first pandemic wave, the series analyzed in this paper begins on 18 March 2020.

The CCC, in which some of the authors of this paper worked during the first wave, was created [12] by the CoM’s Government on 13 March 2020 due to the emergency situation. The CCC aimed to receive daily data from the hospitals, interact with them to filter and debug errors, and use tools that allow their visualization. The information was obtained by means of a spreadsheet in which said hospitals filled out a template shared by all of them on a daily basis, as well as through a web application developed on the Microsoft Teams platform, in which data concerning the situations in the emergency departments were also requested as additional information. For their part, private hospitals sent their daily data in aggregate form only by means of a spreadsheet that had the same template as that used by the public hospitals. The CCC was just in charge of digitizing information; its function was not to make decisions about the management of the hospitals.

### 2.3. Methodology

To achieve the goal of this research, a case study methodology was used because (1) there was an interest in knowing the “how” and “why” of the phenomenon, (2) there was little or no control over behavioral events, and (3) the study’s focus is a contemporary phenomenon—a “case” [13]. Building theory inductively from cases is likely to have important strengths, such as novelty, testability, and empirical validity, which arise from the intimate linkage with empirical evidence” [14], and it is also “likely to produce theory that is accurate, interesting, and testable”, as a wide range of data sources can be used, such as qualitative and quantitative documentations, data, and/or direct observations [15].

Based on the qualitative and quantitative data gathered for the case study, the dynamic response of the healthcare system consisting of the hospital network in the CoM was analyzed. In particular, both the demand and the capacity of the system were studied. System demand refers to the demand or the number of patients that require a service to be provided at a hospital (in this case, the use of a hospital bed combined with treatment and care services) with non-ICU and ICU beds. The availability of non-ICU and ICU beds refers to the readiness or disposal of beds in a hospital that can be offered to potential new patients that demand this service.

In order to analyze the network’s response to the increase in the bed demand, we used the concepts of the bed margin and flexibility in this paper. According to Green [16], hospital occupancy is defined as the ratio of occupied beds to the total number of beds. Following this concept, we defined the bed margin as the ratio of unoccupied beds to the total number of available beds, where the number of unoccupied beds is the difference between the number of available beds and the occupied beds. Note that the proposed bed margin is also related to the well-known concept of utilization in the context of operations management (see, e.g., [17,18]).

The flexibility of a system is not a novel concept. For example, in electric power systems, it is used to characterize the ability of electricity generators to accommodate variation and uncertainty in demand (see, e.g., [19,20]). In this paper, we define the flexibility of a healthcare system as the system’s ability to accommodate a large and unexpected increase in bed demand by modulating the availability of beds across the hospital network over time. Hospitals are managed in order to maintain the balance between quality and costs. Small variations and uncertainties, as well as seasonal fluctuations, in bed demand are managed by hospitals without problems by maintaining an appropriate bed margin. However, dealing with rare abrupt increases in bed demand, such as those generated by the COVID-19 pandemic, indicates a big challenge for hospitals that requires a different type of response—a flexibility induced by the response to a large perturbation.

**Figure 3:**
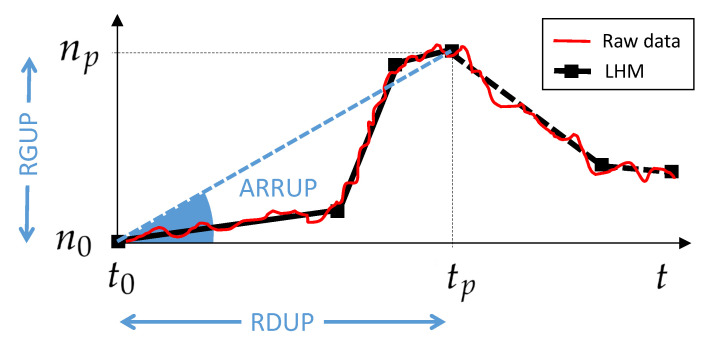
Graphic illustration of the proposed flexibility indicators and their estimation based on the Linear Hinges Model (LHM). The raw data represent the variation in the number of available beds with time, with small random variations around a trend. The LHM is fitted to the raw data in order to estimate the trend, allowing a robust determination of the three indexes.

In order to quantify this flexibility, we propose three different indexes (see Table 2): the Ramp Duration Until the Peak (RDUP), the Ramp Growth Until the Peak (RGUP), and the Average Ramp Rate Until the Peak (ARRUP). According to Figure 3, the RDUP can be easily computed from the raw data as the difference between the time where the peak was reached $t_{p}$ and the starting time of the increase in the bed demand $t_{0}$. In the same way, the RGUP can be obtained from the difference between the number of available beds at the peak $n_{p}$ and at the starting time of the increase in the demand $n_{0}$.

However, to compute the ARRUP index, a robust estimation of the slope of the underlying non-linear bed curve is required. In this paper, we first fit the Linear Hinges Model (LHM) to the raw data, and then directly compute the slopes from the fitted LHM. The LHM was proposed by Sánchez-Úbeda et al. [21]; it is a piecewise linear model defined by *K* knots, the points specifying the pieces (see the illustrative example in Figure 3). Based on the slopes given by the LHM, the ARRUP can be estimated by computing the weighted mean of the slopes just before the peak, where the weights are given by the proportion of the number of days within each segment. Furthermore, both the RDUP and the RGUP can also be estimated directly from the LHM, providing a more robust estimation of these indexes. In this paper, an implementation of the LHM fitting algorithm in the Matlab Software [22] was used.

Note that, although there exist plenty of curve-fitting models that can be used to fit the beds’ curve, such as polynomials or splines, the LHM was selected for estimating the flexibility indicators because it is especially suited for this problem, since it is an efficient approach to curve-fitting under stringent high-noise conditions and thus provides straightforward information on slopes. Moreover, the main advantage of the LHM is that its learning algorithm automatically selects the number and locations of the knots, adapting its complexity to the quality and availability of the data, and thus allows the description of a wide range of functional forms. In particular, the number and positions of the knots are obtained automatically by using a learning algorithm that combines a greedy divide-and-conquer strategy with a computationally efficient pruning approach and special updating formulas [23].

## 3. Results

The results are presented first as an aggregate view of the healthcare system, and second as a disaggregated view by hospital. We distinguish between non-ICU and ICU beds, and apply the proposed bed margin and flexibility indicators in order to quantify the dynamic response.

### 3.1. Aggregate View of the Healthcare System

#### 3.1.1. System Demand for Non-ICU and ICU Beds

**Figure 4:**
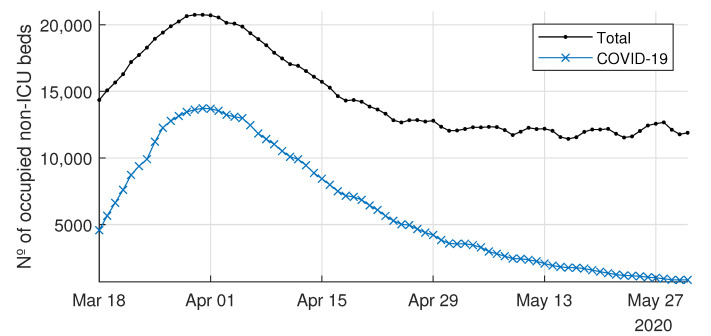
Daily number of total occupied non-intensive care unit (ICU) beds and COVID-19-occupied non-ICU beds.

**Figure 5:**
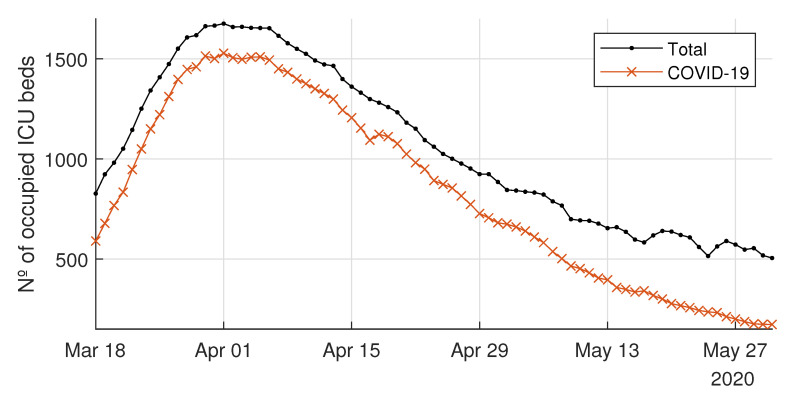
Daily number of total occupied intensive care unit (ICU) beds and COVID-19-occupied ICU beds.

The healthcare system of the CoM was stressed as the number of ill people by SARS-CoV-2 steeply increased in March 2020. Figure 4 shows the daily non-ICU beds occupied by COVID-19 patients for the CoM’s hospitals, as well as the daily total non-ICU occupied beds (i.e., COVID-19 and non-COVID-19). Similarly, Figure 5 shows both the daily ICU beds occupied by COVID-19 patients and the total occupied ICU beds. In the interpretation of the evolution of the curves for each type of bed, it should be taken into account that the average length of stay in an ICU bed was longer than that in a non-ICU bed, and that the flow of patients into ICU beds had a double origin: from the emergency department—in patients with severe clinical presentation—or from non-ICU beds after clinical worsening of the symptoms, which occurred between 2 and 4 days after admission to the hospital [24]. Finally, note that if the patient’s stay in the ICU was successful, they were re-admitted to a non-ICU bed.

This increase in hospital inpatients caused by the new disease implied that the demand for beds in the CoM’s healthcare system grew rapidly during that period. Note that the weekly seasonal pattern, which is visible in the second part of the occupied bed time series (beyond 29 April 2020), was due to the recovery of the regular activity after the peak, where elective patients were usually not admitted on weekends.

From 18 March 2020 to 31 March 2020 (13 days), the daily non-ICU inpatients with COVID-19 increased from 4578 up to 13,725, i.e., an increase of 200%. During the same period, the ICU admissions due to COVID-19 increased by 155% (from 590 to 1502). According to newspapers and television, during the peak period, the time spent by patients in emergency rooms and hallways waiting for a bed increased dramatically in many hospitals of the CoM (see, e.g., [25]).

#### 3.1.2. Availability of Non-ICU and ICU Beds

Creating enough capacity in the CoM’s hospitals to deal with the COVID-19 demand was a big challenge during March 2020. This capacity expansion meant not only increasing the availability of physical beds, but also of specialized personnel and the required equipment.

In order to fulfill this objective, hospitals implemented several strategies. At first, operating rooms and semi-intensive care beds were transformed into fully operating ICU beds within existing hospitals. Later, several hotels were turned into recovery units to which non-critical patients could be transferred. A field hospital was also opened on 22 March 2020 in an existing convention center (IFEMA) to release some of the pressure on permanent hospitals. This hospital started with 185 non-ICU beds and reached a maximum at 3 April 2020 with 1150 and 10 non-ICU and ICU beds, respectively. It was operative until 30 April 2020. Furthermore, from the demand side, the Spanish government implemented the confinement of the country with severe restrictive measures to lower the spread of the virus and to reduce the demand for beds in the short term, i.e., the so-called “flattening the curve” [26,27].

**Figure 6:**
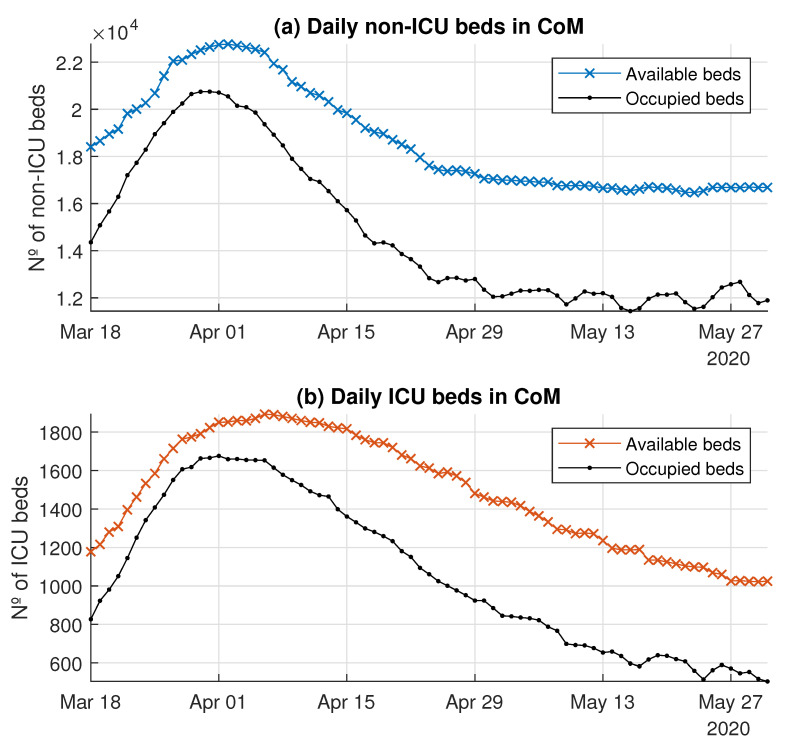
Daily availability and occupation of beds in the CoM’s hospital system.

Figure 6 compares the system’s capacity and the demand for non-ICU and ICU beds. This capacity is measured as the number of available beds. The numbers of non-ICU and ICU beds provided by the system rose drastically to follow the growth of the demand for beds.

#### 3.1.3. System Bed Margin

**Figure 7:**
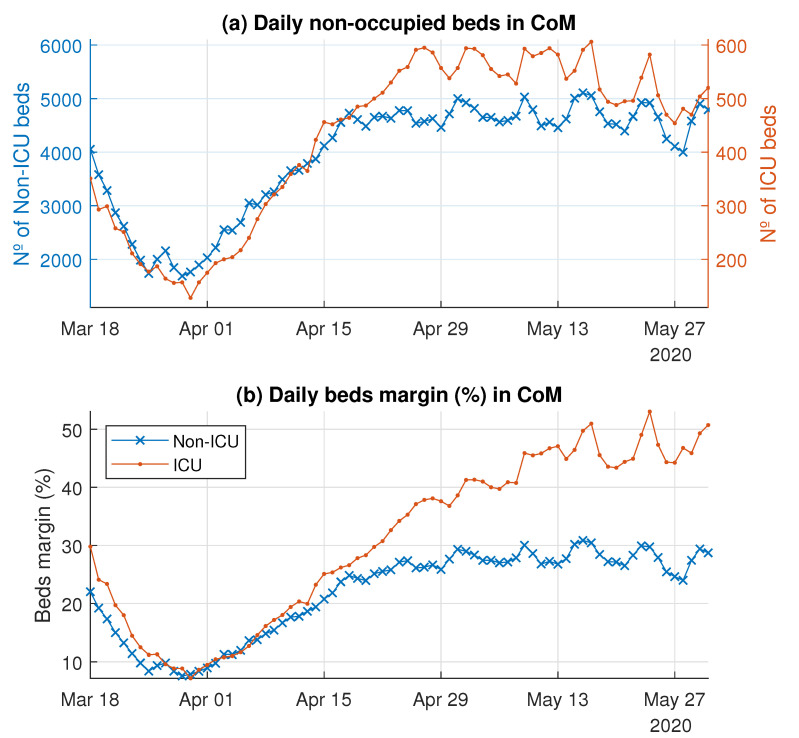
Daily unoccupied beds and the bed margin (%) in the CoM’s hospital system.

Figure 7 shows the daily number of unoccupied beds and the bed margin for both the non-ICU and ICU beds. On 18 March 2020, at the beginning of the peak, the number of unoccupied non-ICU beds was 4053, whereas the number of unoccupied ICU beds was 351, making the bed margins 22% and 29.8%, respectively. However, 29 March 2020 was the worst day in terms of the non-ICU bed margin (7.6%) for the health system as a whole; the number of unoccupied non-ICU beds reached a minimum value of 1688. For the ICU beds, the worst day was 30 March 2020, with 128 unoccupied ICU beds, which represents an ICU bed margin of 7.1%. From 1 April 2020, the margin started to recover. In particular, the non-ICU bed margin stabilized in May, with an average value of 27.3% (4664 unoccupied beds). On the other hand, the ICU bed margin continued increasing up to 50% (around 500 unoccupied beds).

#### 3.1.4. Healthcare System Flexibility

**Figure 8:**
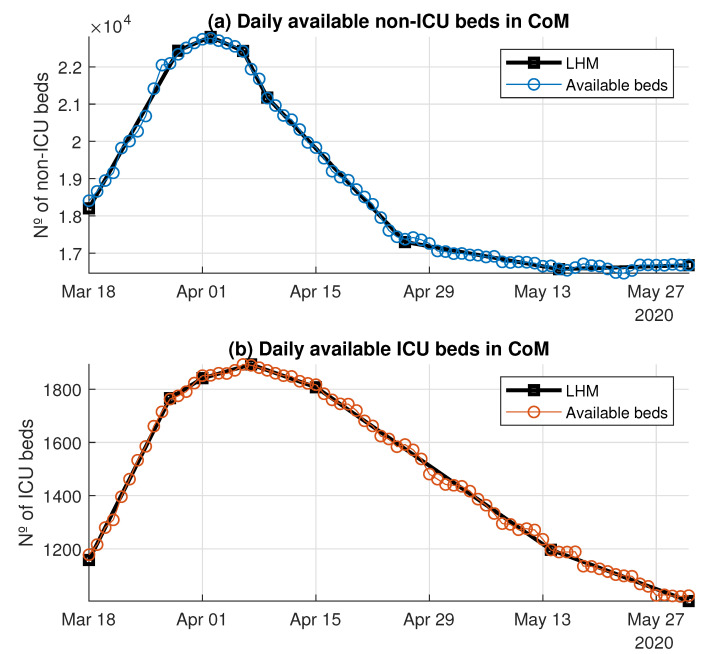
Model of the available beds using LHM.

Figure 8 shows the LHM obtained for the available non-ICU and ICU beds. The LHM for the non-ICU beds consisted of eight knots, whereas the model for the ICU beds had K=7.

According to the LHM for non-ICU beds (Figure 8, top), the peak was on 2 April 2020, when the maximum number of available non-ICU beds was reached. This model detected two different ramps until the peak (see Table 3). In the first period, the slope was around 384 beds/day over 11 days. Just four days, before the peak, during the second segment given by the model, the ramp rate went down to around 92 non-ICU beds/day. Something similar happened for the available ICU beds, where the slope also slowed down just before the peak (Figure 8, bottom). In this case the LHM estimated three segments until 7 April 2020 (see Table 4), with a slope of nearly 61 ICU beds/day during the first 10 days. The duration of the third ramp was six days, with a rate of around 9 beds/day. The reduction with time in the slopes of both non-ICU and ICU available beds confirmed the great efforts during the first days to increase the bed capacity, as well as the difficulties in obtaining additional capacity near the peak.

Based on these LHMs, the flexibility indicators were estimated (see Table 3 and Table 4). In particular, the ARRUP for non-ICU beds was 305.56 beds/day (384.47×0.73+92.22×0.27), the RDUP was 15 days, and the RGUP was 4598 beds. For the ICU beds, the ARRUP was 36.73 beds/day, the RDUP was 20 days, and the RGUP was 735 beds.

### 3.2. Disaggregated View by Hospital

In the previous section, the system’s response to the COVID-19 pandemic was analyzed. This provided a global view of the aggregated response of the CoM’s hospital network as a unique system. However, not all the hospitals were confronted with the same bed demand, nor did they provide the same flexibility in order to increase the bed capabilities. In this section, we provide a disaggregated view by hospital. In order to quantify the response of each hospital, the concepts of the bed margin and flexibility were applied straightforwardly to each hospital.

#### 3.2.1. Demand and Availability of Beds by Hospital

**Figure 9:**
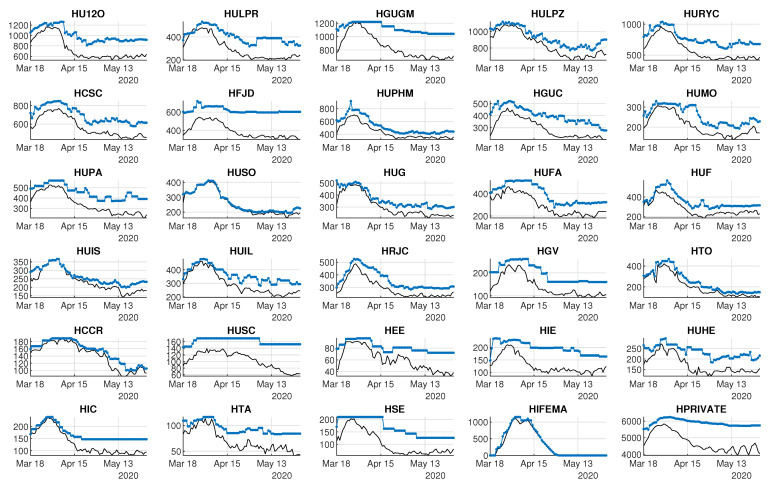
Capacity and demand for non-ICU beds by hospital. Black lines represent occupied beds. Colored lines represent available beds.

**Figure 10:**
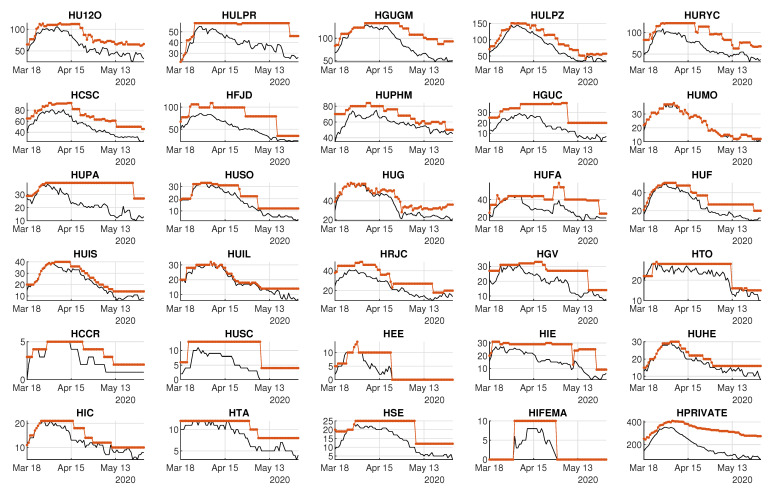
Capacity and demand for ICU beds by hospital. Black lines represent occupied beds. Colored lines represent available beds.

Figure 9 shows the daily number of occupied non-ICU beds by hospital, whereas Figure 10 shows the demand for ICU beds for each hospital. The demand for beds at HIFEMA, the hospital that was available on 22 April 2020, was different from the rest because it was created specifically to release some of the pressure on permanent hospitals during the critical days of the peak. Logically, the daily bed demand of a given hospital is limited by its daily availability of beds.

Concerning the availability of beds, Figure 9 and Figure 10 also show the daily numbers of available non-ICU and ICU beds, respectively. Note that most of the hospitals were able to increase their bed capacity during the critical days, although some of them had more difficulties with maintaining a reasonable bed margin.

#### 3.2.2. Bed Margin by Hospital

**Figure 11:**
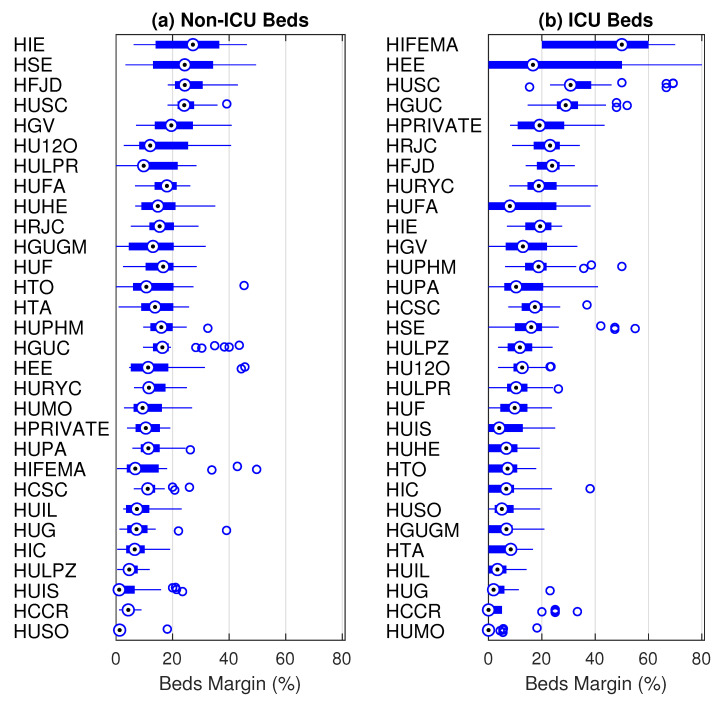
Compact boxplot representation of the non-ICU and ICU bed margins (%) by hospital. For each distribution, the median is represented by a circled dot, the interquartile range by a rectangle, the whiskers by lines, and the outliers by empty circles.

From 18 March 2020 to 15 April 2020 the system’s margin was lower than 30% for both types of beds (see Figure 7). In particular, the daily minimum bed margins were 7.6% and 7.1% for non-ICU and ICU beds, respectively. However, during this period, the bed margins varied from hospital to hospital. Figure 11 shows a boxplot of the bed margins for each hospital computed for this period of 29 days. The hospitals are sorted according to the third empirical quartile for each type of bed.

For non-ICU beds (Figure 11, left), the margin was lower than 60% for all hospitals. In fact, all but seven hospitals had a non-ICU bed margin lower than 25% for at least 75% of the days, i.e., the third quartile of the bed margin was lower than 25%. HUSO was the most stressed hospital in terms of non-ICU beds during this period, with a median bed margin of 1.3% (five beds). Concerning ICU beds, the lack of unoccupied beds in several hospitals over several days is obvious. Table 5 shows the hospitals where the ICU bed margin was lower than or equal to 15% for at least 75% of the days, i.e., those where the third quartile of the bed margin was lower than 15%. Furthermore, the second quartile of the number of unoccupied ICU beds was equal to 1 for many hospitals, i.e., for half of the days, no more than one ICU bed was unoccupied in those hospitals. HUMO and HCCR did not have unoccupied ICU beds for at least 75% of the days.

#### 3.2.3. Flexibility by Hospital

**Figure 12:**
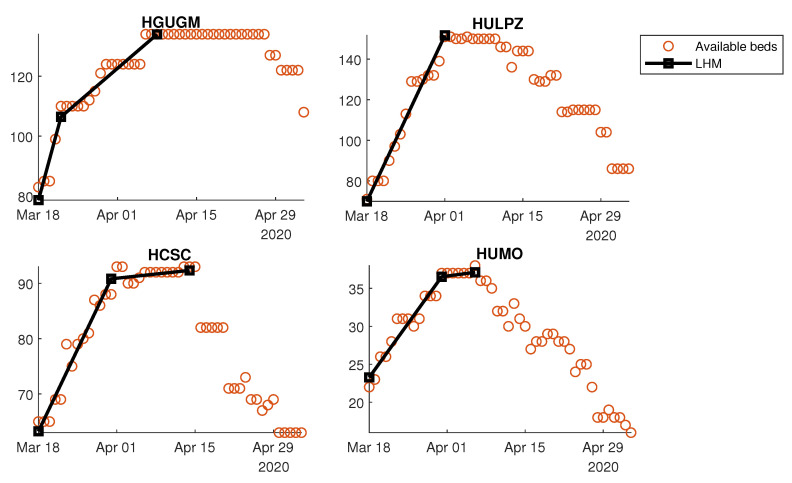
Model of the number of available ICU beds using the LHM (details of four hospitals).

It was possible to calculate the previously defined indexes—RDUP, RGUP, and ARRUP (see Table 2)—for each hospital using the same approach based on the LHM. In this case, it was even more necessary to have a robust slope estimation mechanism because the random variations around the main curve were more important. For example, Figure 12 shows the details of the LHM fitted to the available ICU beds of four different hospitals. Notice that the LHM allows the extraction of the main underlying response for each particular hospital by assimilating the daily fluctuations in the original data.

Table 6 shows the estimated values of the indexes for the non-ICU beds for each hospital, whereas Table 7 shows the results for the ICU beds. According to the first table, larger public hospitals, such as HU120, HGUGM, and HULPZ, which had more than 1000 regular beds on 18 March 2020, had a lower RGUP and ARRUP than other medium-sized hospitals, such as HUF, HRJC, or HUPHM. For example, HU120 increased the number of available non-ICU beds by 188 (10%), whereas the HUF was able to increase by 241 non-ICU beds (70%). However, according to Table 7, the HULPZ was the public hospital with the largest RGUP in ICU beds, as it was able to increase by 82 ICU beds (120%) in 15 days. HU12O and HGUGM also had a large RGUP.

## 4. Discussion

Analyzing healthcare data through the first wave of SARS-CoV-2 has brought up an opportunity to better understand hospital performance under these severe circumstances. Specifically, by analyzing the flexibility and bed margin, it is possible to dynamically graph—as an alternative or complement to other, more static models—not only the day-to-day response of the system as a whole for all of the hospitals within the CoM region, but also the response of each hospital (Figure 9 and Figure 10). These graphs provide information for hospital managers and healthcare system supervisors, since they can check (in a dynamic way) certain situations that are referred to later on.

This paper focuses on modeling the hospital capacities in terms of ICU and non-ICU beds during the pandemic while distinguishing between patients with and without COVID-19, since most publications focus on bed management in other contexts [28,29,30,31,32,33] (except for Condes and Arribas [34] and Fanelli et al. [35]). We have not found a work in the literature that gives information on this topic with the details and large number of hospital and inpatients that this article uses, highlighting the fact that the hospitals involved in this study were in the epicenter of the first wave of the COVID-19 pandemic at the European level. Therefore, the data presented here could be beneficial in the future in order to compare what happened during the first wave in other healthcare systems and to better prepare in the event new epidemic waves caused by SARS-CoV-2 or other pathogens. Finally, this article shows a way to classify hospitalized patients in pandemic situations and to manage infected and uninfected inpatients. Among other things, this classification makes it possible, with the previously mentioned safeguards, to compare the first wave with the pre-pandemic situation in order to know how many patients could not be admitted to hospitals for diseases other than COVID-19. The previous situation, in our opinion, should be a clear invitation to other researchers to address such a critical scenario when that information is available.

The above-mentioned achievements were obtained by analyzing and extracting information from the available data from the CCC. The relevant figures of the first pandemic wave in the CoM were: From 18 March 2020 to 31 March 2020 (13 days), COVID-19 inpatients increased by 200% in non-ICU wards and by 155% in ICU wards. The maximum occupancy of available beds took place on 31 March 2020. On that day, in all of the hospitals that we analyzed, the total number of available beds was 23,463, of which 21,549 (92%) were occupied. The capacity in many hospitals was exhausted, but the fear that the demand would continue to increase caused the number of available beds to increase by 160 in 48 h, reaching the figure of 23,623 beds on 2 April 2020, with an occupancy of 90.3%.

The previous paragraph describes the first wave scenario in the CoM. Our analysis, which was focused on the proposed bed margin and flexibility indexes, shows that hospitals adapted to unexpected requirements with different responses. Notice the following fact: All but seven hospitals presented a non-ICU bed margin (Figure 11, left) lower than 25% for at least 75% of the days, and one of them presented a median bed margin of 1.3% (five beds). Focusing on ICU beds, the lack of unoccupied beds in several hospitals over several days was notorious (Table 5). For at least 75% of the days, in 14 of the 30 hospitals analyzed, the ICU bed margin was lower than or equal to 15%, and two hospitals lacked free ICU beds. Occupied non-ICU beds and ICU beds did not return to the values of 18 March 2020 until the third week of April and the first week of May, respectively.

Under normal conditions, hospitals sometimes operate with difficulties, especially in emergency departments. Contrary to popular belief, hospitals are complex but vulnerable institutions [36]. This vulnerability is mainly due to three factors: First, as seen with personal protective equipment and laboratory reagents, they are highly dependent on external supplies. Second, the key staff is highly specialized and is not easy to increase or replace. Third, their infrastructure (beds, doctor offices, medical equipment, etc.) is dimensioned to care for a range of pathologies and a maximum number of patients. Therefore, a modest variation in the admission volume, as happens when the seasonal flu arrives, can lead the hospital beyond its functional reserves.

It is worth noting how unexpected demands cause situations like those shown in Figure 4 and Figure 5. These figures compare the beds occupied by patients with or without COVID-19 who were hospitalized inside and outside ICUs. Moreover, they show how the demand for hospitalizations due to COVID-19 severely reduced the hospitals’ capacities to admit new patients with other types of pathologies, especially in the ICUs. Flexibility in available beds is a significant driver for managing hospitals efficiently, and its importance acquires greater relevance in situations such as the COVID-19 pandemic.

Quantifying the flexibility with a regression technique such as the LHM is an attempt to model the dynamic performance from the observed data. More specifically, the LHM was used to obtain a robust estimation of the proposed flexibility indexes by means of the piecewise slope estimations that it provided for the time series of the number of available beds. In particular, the LHM obtained for available non-ICU and ICU beds in the CoM’s whole health system detected several different ramps before and after the peak (see Figure 8). The reduction of the available beds after the peak is explained easily by the systematic decrease in bed demand. However, the slowdown in the ramp rate of available beds started before the bed demand decreased (see Figure 5). The systematic reduction in the bed margin before the peak is noticeable for both non-ICU and ICU beds (see Figure 6); thus, the ramps began to slow down as they approached the peak. The justification is supported by the reduction in bed demand and the system’s limitations for additional capacity when the bed demand was peaking. These limitations show up due to the lack of extra physical space, equipment, and qualified personnel.

The flexibility performance indicators and the LHM model were used in an attempt to provide insights that could help improve the hospitals’ management and decision making. However, it cannot be determined a priori what the optimal level of beds or human resources for facing a pandemic crisis should be. Unfortunately, it is not possible to respond to this question until after a new epidemic wave is addressed.

Figure 9 and Figure 10 show the supply of and demand for beds. The observed variations can be explained, in a summarized way, by saying that hospitals of high complexity, which are those with more beds, generally fared better than hospitals of lower complexity (which are the ones with the fewest beds). This fact emphasizes the importance of having a real-time control panel that could avoid having hospitals under the pressure of a very narrow bed margin. Distributing inpatients throughout the hospital system can ease the pressure on some hospitals. In this way, two main objectives will be achieved in a very complex environment: avoiding the professionals’ exhaustion and providing the best care to patients. Let us remember that both objectives are intimately interweaved.

This paper focuses on the first wave of the COVID-19 pandemic in a specific geographic area (the CoM in Spain). The proposed model is dynamic (compared to existing static models) and could be used in other geographic regions, as well as in future waves of pandemics or other health crises. The introduction of new mathematical models for emulating the dynamics of a hospital network in terms of capacity, flexibility, and bed margin will pave the way to a better understanding of the impacts of health policies and resource management during health crises. An example of this type of research is the work by Wood [37], where the ICU capacity of a large British public hospital in the context of COVID-19 was analyzed using public data from the Intensive Care National Audit and Research Centre [38].

## 5. Conclusions

The main objective of our study was to analyze the dynamic response of the CoM’s hospitals during the first wave of the SARS-CoV-2 pandemic. In order to carry out this analysis, a set of very informative indicators was proposed. These indexes were able to summarize the observed dynamics of the curve of the available beds during the pandemic in a simple way. In particular, we proposed the bed margin as a useful index of the available resources, as well as three new indicators for capturing the flexibility with respect to the growing demand for beds. The main indicator of flexibility is the Average Ramp Rate Until the Peak (ARRUP), but it cannot be interpreted in isolation. The Ramp Duration Until the Peak (RDUP) and the Ramp Growth Until the Peak (RGUP) provide useful information that allows one to answering questions, such as: For how many days can the observed ramp rate be maintained? What is the maximum number of beds that can be available?

Our results, which are based on the proposed bed margin and flexibility indexes, show that the hospital network in the CoM can increase the number of available ICU beds with a rate of at least 61 beds/day for 10 days. Furthermore, the considered hospitals were able to adapt to unexpected requirements with different responses. This study offers valuable insights concerning not only the system’s response, but also about the individual responses of the CoM’s hospitals.

The proposed indicators are ready to use for monitoring of networks of hospitals. For example, the estimated indexes for a particular hospital during the first pandemic wave can be used as a reference for this hospital, which is useful for monitoring its behavior during a similar pandemic wave. Future research could apply the proposed methodology based on the bed margin and flexibility indexes in order to analyze a health system’s response during other pandemic waves or similar catastrophes. The comparison of our findings during this first wave with those of future responses may provide additional insights. Moreover, due to the existence of particular characteristics of the hospitals that could affect their dynamic responses under these critical circumstances, future research may be developed in order to identify machine learning models that are able to estimate a given hospital’s flexibility as a function of its characteristics.

## Figures and Tables

**Table 1:** List of the Community of Madrid’s (CoM’s) hospitals and their main characteristics.

ID	Hospital	Complexity Level	N° of Non-ICU Beds ^a^	N° of ICU Beds ^a^
1	HU12O	High	1116	79
2	HULPR	High	387	20
3	HGUGM	High	1046	108
4	HULPZ	High	1137	35
5	HURYC	High	773	67
6	HCSC	High	717	61
7	HFJD	High	507	18
8	HUPHM	High	518	49
9	HGUC	Medium	403	19
10	HUMO	Medium	285	15
11	HUPA	Medium	442	14
12	HUSO	Medium	344	13
13	HUG	Medium	400	33
14	HUFA	Medium	374	12
15	HUF	Medium	344	10
16	HUIS	Medium	295	8
17	HUIL	Medium	358	8
18	HRJC	Medium	363	3
19	HGV	Medium	195	9
20	HTO ^b^	Medium	180	16
21	HCCR	Medium	152	0
22	HUSC	Medium	103	4
23	HEE	Low	75	0
24	HIE	Low	168	4
25	HUHE	Low	243	10
26	HIC	Low	159	9
27	HTA	Low	91	6
28	HSE	Low	134	6
29	HIFEMA ^c^	Field hospital	1150	10
30	HPRIVATE ^d^	Medium/low	5523	362

^a^ Average number of available beds during January and February 2020; ^b^ Average number of available beds during January and February 2019; ^c^ Number of available beds on 3 April 2020 when it was fully operative (opened at 22 March 2020); ^d^ Number of available beds on 18 March 2020.

**Table 2:** Proposed flexibility indexes.

Index	Description	Units
RDUP	Ramp Duration Until the Peak	days
RGUP	Ramp Growth Until the Peak	beds
ARRUP	Average Ramp Rate Until the Peak	beds/day

**Table 3:** Estimated slopes for available non-ICU beds.

Starting Date	End Date	Days	Slope	Weight
18 March 2020	29 March 2020	11	384.47	0.73
29 March 2020	2 April 2020	4	92.22	0.27

**Table 4:** Estimated slopes for available ICU beds.

Starting Date	End Date	Days	Slope	Weight
18 March 2020	28 March 2020	10	60.77	0.5
28 March 2020	1 April 2020	4	18.78	0.2
1 April 2020	7 April 2020	6	8.64	0.3

**Table 5:** Quartiles (1 to 3) of the bed margin (%) and number of unoccupied ICU beds for hospitals. Q1, Q2, and Q3 are the three quartiles.

Hospital	ICU Bed Margin			Unoccupied ICU Beds		
	Q1	Q2	Q3	Q1	Q2	Q3
HUMO	0.00	0.00	1.09	0	0	0
HCCR	0.00	0.00	5.00	0	0	0
HUG	1.27	1.89	5.94	1	1	3
HUIL	0.00	3.33	6.67	0	1	2
HTA	0.00	8.33	8.33	0	1	1
HGUGM	0.00	6.72	8.99	0	8	11
HUSO	2.34	5.00	9.38	1	1	3
HIC	0.00	6.67	9.52	0	1	2
HTO	0.00	7.14	10.71	0	2	3
HUHE	0.00	6.67	10.81	0	1	3
HUIS	0.00	4.00	12.85	0	1	5
HUF	4.45	9.80	14.58	2	5	6
HULPR	6.96	10.34	14.59	4	6	8
HU12O	9.42	12.61	14.64	9	12	15

**Table 6:** Non-ICU bed flexibility by hospital. The initial and the maximum numbers of available beds, as well as the three flexibility indicators, are shown for each hospital. The top eight hospitals have a high complexity (see Table 1).

	Available Beds		Flexibility		
Hospital	Ini	Max	RGUP	RDUP	ARRUP
	Beds	Beds	Beds	Days	Beds/Day
HU12O	1069	1257	188	21	9.4
HULPR	388	529	141	14	10.8
HGUGM	1112	1223	111	12	10.1
HULPZ	1034	1112	78	9	9.8
HURYC	802	1024	222	12	20.2
HCSC	712	867	155	16	10.4
HFJD	577	680	103	11	10.2
HUPHM	571	835	264	11	26.3
HGUC	446	522	76	13	6.4
HUMO	259	330	71	13	5.9
HUPA	488	569	81	15	5.8
HUSO	312	418	106	19	5.9
HUG	487	498	11	15	0.8
HUFA	408	516	108	14	8.4
HUF	328	569	241	16	16.0
HUIS	290	368	78	18	4.6
HUIL	346	481	135	14	10.4
HRJC	298	521	223	12	20.3
HGV	195	260	65	14	5.0
HTO	288	460	172	17	10.7
HCCR	160	190	30	27	1.1
HUSC	139	170	31	12	2.8
HEE	78	97	19	20	1.0
HIE	149	237	88	3	43.7
HUHE	262	305	43	15	3.0
HIC	175	240	65	12	5.9
HTA	103	116	13	19	0.7
HSE	205	213	8	29	0.3
HIFEMA	1	1155	1154	17	72.1
HPRIVATE	5446	6228	782	15	55.9

**Table 7:** ICU bed flexibility by hospital. The initial and the maximum numbers of available beds, as well as the three flexibility indicators, are shown for each hospital. The top eight hospitals have a high complexity (see Table 1).

	Available Beds		Flexibility		
Hospital	Ini	Max	RGUP	RDUP	ARRUP
	Beds	Beds	Beds	Days	Beds/Day
HU12O	61	112	51	34	1.6
HULPR	22	58	36	11	3.6
HGUGM	79	134	55	22	2.6
HULPZ	70	152	82	15	5.8
HURYC	78	123	45	13	3.8
HCSC	63	92	29	28	1.1
HFJD	66	103	37	9	4.6
HUPHM	69	83	14	22	0.7
HGUC	24	39	15	49	0.3
HUMO	23	37	14	20	0.7
HUPA	28	39	11	14	0.9
HUSO	17	33	16	14	1.3
HUG	40	58	18	9	2.2
HUFA	31	60	29	45	0.7
HUF	22	52	30	15	2.1
HUIS	17	41	24	26	1.0
HUIL	18	30	12	26	0.5
HRJC	41	49	8	18	0.5
HGV	28	34	6	33	0.2
HTO	21	28	7	10	0.8
HCCR	3	5	2	20	0.1
HUSC	6	13	7	7	1.2
HEE	4	13	9	15	0.6
HIE	19	30	11	3	5.5
HUHE	14	30	16	19	0.9
HIC	12	21	9	10	1.1
HTA	12	12	0	0	0.0
HSE	19	25	6	14	0.5
HIFEMA	0	10	10	17	0.6
HPRIVATE	253	409	156	23	7.1

## Data Availability

Restrictions apply to the availability of these data. The data were obtained from the network of public and private hospitals in the CoM and are available from the CCC with the permission of the Department of Health, CoM Government. The dataset is neither public nor available in the way that it has been used as a source in this article.

## Software Availability Statement

The LHM fitting software used in this study is available from IIT http://www.iit.comillas.edu/, but restrictions apply to the availability of the software, which is not publicly available.

## Abbreviations

The following abbreviations are used in this manuscript:

AC	Autonomous Community
ARRUP	Ramp Duration Until the Peak
CCC	COVID-19 Control Center
CoM	Autonomous Community of Madrid
COVID-19	Coronavirus Disease 2019
CS	*Consejería de Sanidad*—Departments of Health
DEA	Data Envelopment Analysis
ICU	Intensive Care Unit
LHM	Linear Hinges Model
non-ICU	Non-Intensive Care Unit
RDUP	Ramp Duration Until the Peak
RGUP	Ramp Growth Until the Peak
SARS-CoV-2	Severe Acute Respiratory Syndrome Coronavirus 2
SNS	*Sistema Nacional de Salud*—Spanish National Health System
